# Undifferentiated Endometrial Sarcoma of the Ovary: A Case Report with Review of Recent Literature and Discussion of Lacking Specificity of CD10 Immunoreactivity

**DOI:** 10.4061/2010/608519

**Published:** 2009-10-21

**Authors:** Hermann Brustmann, Ingrid M. Geiss, Susanne Hinterholzer

**Affiliations:** ^1^Department of Pathology, Landesklinikum Thermenregion Mödling, Sr. M. Restitutagasse 12, Mödling A-2340, Austria; ^2^Department of Obstetrics and Gynecology, Landesklinikum Thermenregion Mödling, Sr. M. Restitutagasse 12, Mödling A-2340, Austria

## Abstract

Undifferentiated endometrial sarcomas (UESs) of the ovary are very rare tumors. This paper presents a case of a 56-year-old patient with a history of hysterectomy and bilateral salpingectomy seven years ago for uterine leiomyomata. Intraoperatively, a tumor originating from the left ovary, adherent to the sigmoid colon, with infiltration of the small intestine and the vaginal apex was found. Histologically, the tumor was composed of pleomorphic round and oval to spindled cells with polymorphous vesicular nuclei with coarse chromatin and large nucleoli. Mitotic activity was brisk. There were large necrotic areas. Adjacent to the tumor tissue endometrium-like glands surrounded by fibrous stroma with macrophages corresponding to ovarian endometriosis were noted. Tumor cells showed diffuse strong immunoreactivity for vimentin and patchy strong staining for CD10; no reactivities were found for AE1/AE3, desmin, S-100, LCA, CD20, c-kit, and CD31. The patient died of her neoplastic disease four months postoperatively. CD10 is frequently expressed in different gynecopathological as well as other lesions, and, thus, nonspecific without relevance to the classification of this case. Morphological features, extensive sampling, and appropriate immunohistochemistry including markers for cytokeratins and myogenic differentiation are mandatory to arrive at the correct diagnosis.

## 1. Introduction

Ovarian endometrioid stromal sarcomas (ESSs) are rare tumors with about 50 cases reported in the literature. They are composed of cells resembling the stromal cells of normal proliferative endometrium. These tumors are reported at any age, but most of them occur in the fifth and sixth decades. At presentation, the symptoms are nonspecific and attributable to the presence of a pelvic mass. At the time of operation, most of ovarian ESS are high stage [1–6].

Previously, ESSs in general and in the ovary were categorized in low and high grade tumors based on mitotic counts. High grade ESS of the ovary accounted for 17% of cases only in one study [4, 5]. However, the lack of specific evidence of endometrial stromal cell origin in most cases of high-grade tumors leads to the designation of undifferentiated endometrial sarcomas (UESs). These sarcomas are characterized by marked cellular pleomorphism and brisk mitotic activity and carry a very poor prognosis [7, 8]. CD10, the common acute lymphoblastic lymphoma antigen (CALLA), has been reported on as a marker for normal and neoplastic endometrial stromal cells previously [9, 10]. Recently, the diagnostic consideration of CD10 immunoexpression in endometrial stromal neoplasms has changed significantly [7]. In this study we describe the clinicopathologic features of a UES of the ovary with regard to recently published literature and emphasis on a discussion of lacking relevance of CD10 immunoreactivity in the differential diagnosis.

## 2. Case Presentation

A 56-year-old patient presented with a tumor of the left ovary, which was found during abdominal sonography. She noted an increase of her abdomen associated with a feeling of swelling. Her history was remarkable for hysterectomy and bilateral salpingectomy seven years ago for uterine leiomyomata. Gynecological examination showed a tumor filling the pelvis minor. Computed tomography revealed a 12 × 9 cm partially solid, partially cystic lesion of adnexal origin; no enlarged lymph nodes were identified.

Intraoperatively, a tumor originating from the left ovary and adherent to the sigmoid colon, the small intestine, and the vaginal apex was found in the pelvis minor. The right ovary was unremarkable. Tumor, vaginal apex, omentum majus, a segment of the small intestine as well as right ovary were removed; there were no ascites and no clinical impression of residual tumor.

The tumor was submitted for frozen section examination, and a diagnosis of an undifferentiated ovarian neoplasia was given. The resected specimens were fixed in 10% neutral buffered formaldehyde solution. The tumor was surrounded by a smooth capsule, which showed broad defects. The cut surface consisted of gray-yellowish friable and partially necrobiotic tissues. Stainings were carried out on sections of the paraffin-embedded tissue blocks cut at 3 *μ*m. Besides hematoxylin and eosin staining (H&E), a standard immunohistochemical testing was conducted using a BenchMark series automated slide stainer (Ventana Medical Systems) with commercially available antibodies form DAKO (Carpinteria, CA) to the cytokeratin marker AE1/AE3 (1 : 50), desmin (1 : 50), vimentin (prediluted, rediluted at 1 : 5), MIB-1 (1 : 100), LCA (prediluted), S-100 (1 : 200), CD20 (1 : 4) as well as prediluted ready-to-use antibodies from Ventana to c-kit, synaptophysin, estrogen- and progesterone receptor, CD31 and CD10. Additionally, a reticulin-staining after Gömöri was performed.

**Figure 1:**
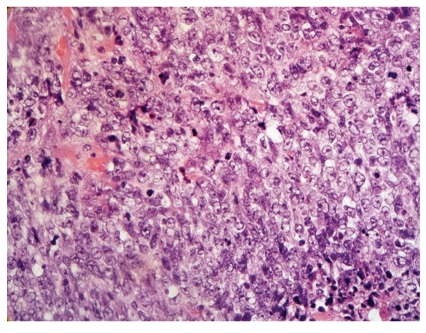
The high grade UES of the ovary is composed of dedifferentiated round and oval to spindled cells. The nuclei are polymorphous; vesicular with coarse chromatin and large nucleoli; the cytoplasmata are scant. Mitotic figures are readily identified (H&E, ×400).

**Figure 2:**
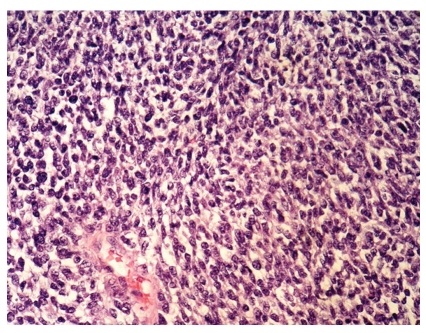
Areas with smaller and more monotonous cells are observed focally (H&E, ×400).

**Figure 3:**
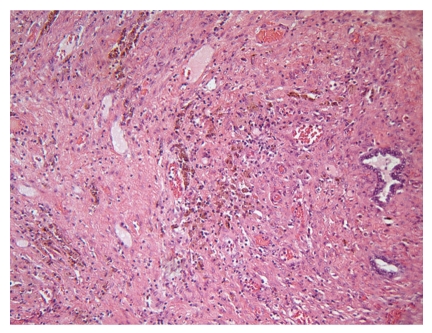
Endometrium-like glands corresponding to ovarian endometriosis were surrounded by broad fibrous stroma with macrophages (×100).

Histologically, the tumor was composed of pleomorphic round and oval to spindled cells. Their nuclei were polymorphous vesicular with coarse chromatin and large nucleoli (Figure 1). The cytoplasmata were scant. More than 10 mitotic figures per 10 high power fields were readily identified. Fibrous septa intersected the tumor nodules. Geographically confluent necrotic areas were abundant. A network of interstitial thin walled blood vessels was demonstrated by CD31 immunohistochemistry. Reticulin fibers surrounded single tumor cells. There were transitions to areas with rather monotonous cells (Figure 2). Call-Exner bodies were not identified. Tumor cells infiltrated the ovarian capsule were demonstrated on its surface, and infiltrated blood as well as lymphatic vessels. Adjacent to the tumor tissue endometrium-like glands corresponding to ovarian endometriosis were found, surrounded by broad fibrous stroma with macrophages; there was no condensation of tumor cells around endometriotic glands “periglandular collaring” or polypoid intraluminal projections by the sarcoma (Figure 3). 

**Figure 4:**
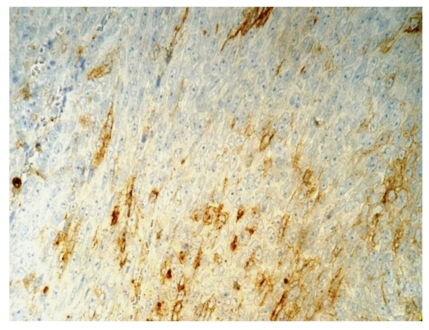
Tumor cells of high grade ovarian UES show focal strong immunostaining for CD10 (×400).

Immunohistochemically, tumor cells showed diffuse strong reactivity for vimentin and patchy strong staining for CD10 in about 50% of cells (Figure 4); there was no staining of tumor cells for AE1/AE3, desmin, S-100, LCA, CD20, c-kit, and CD31. Estrogen and progesterone receptor reactivities were noted focally in a small percentage of neoplastic cells only. In some tumor areas, up to 60% of tumor cells reacted for MIB-1. Endometriotic glands showed abundant nuclear immunostaining for hormone receptors. 

There was histological evidence of tumor infiltration in the resected specimens of the vaginal apex and the segment of the small intestine with microscopically positive margins at the latter. The right ovary as well as the omentum majus was free of tumor. The sections of the previous hysterectomy specimen were reviewed; they showed benign leiomyomata and discrete foci of adenomyosis without architectural or cytological atypia, and there was no evidence for sarcomatous changes.

Based on these findings the tumor was interpreted as high-grade ESS or UES, respectively, of the ovary with infiltration of the vaginal apex and the small intestine.

There was no postoperative adjuvant therapy. A second-look laparotomy two months later was done due to a CT scan showing an intestinal mass and revealed a conglomerate tumor of 10 × 10 cm, involving small and large intestine. This tumor was biopsied only and was histologically identical to the previously diagnosed UES. The patient was referred to an oncological center for radiation therapy and died four months postoperatively of her neoplastic disease.

## 3. Discussion with Review of the Literature

The common acute lymphoblastic leukemia antigen (CALLA or CD10), a 90 to 110-kDa membrane-bound endopeptidase, is expressed on the cell surface of most cases of acute lymphoblastic leukemia, other types of leukemia, as well as lymphomas and nonhematopoietic neoplasms [11, 12]. This cell surface enzyme reduces cellular response to peptide hormones by regulating local peptide concentration [11]. Thus, many hormone-sensitive and peptide-sensitive cells as well as their corresponding neoplasms express CD10 antigen [11], including normal endometrial stroma and ESS [9, 10].

Although CD10 has been considered a marker for ESS [11], some studies have shown that many other uterine neoplasms like uterine smooth muscle tumors, adenosarcomas, malignant Müllerian mixed tumors, rhabdomyosarcomas, endometrial carcinomas, endocervical adenocarcinomas, uterine tumors resembling ovarian sex cord tumors, perivascular round cell tumors, mesonephritic carcinomas, and gestational trophoblastic disease may express CD10 [12]. In the ovary, Ordi and Romagosa [13] noted a very limited but strong CD10 positivity in ovarian stroma. In contrast, Khin and Kikkawa [14] and Groisman and Meir [15] detected no immunoreactivity for CD10 in stromal cells of normal ovaries, suggesting that CD10 may help in identifying subtle foci of endometriosis surrounding Müllerian-type glands as endometrial stroma stains for CD10. However, Oliva and Garcia-Miralles [12] noticed focal CD10 expression in ovarian stroma being stronger in cases with a background of stromal hyperthecosis or a presence of corpora lutea questioning the use of CD10 when focally present in stroma surrounding Müllerian-type glands. There is no evidence for CD10 expression in ovarian surface epithelial cells or epithelial inclusions [13–15]. Nevertheless, CD10 may be positive in serous and mucinous carcinomas and Brenner tumors as well as the stroma surrounding serous borderline tumors and serous, endometrioid, and clear cell carcinomas [12–14]. Oliva et al. [12] reported on CD10 expression in a large series of pure stromal and sex cord-stromal tumors of the ovary. They observed that frequency and intensity of CD10 immunoreactivity in these tumors are low and contrast with the typical strong and diffuse immunostaining in endometrial stromal tumors, and concluded that CD10 should not be used in isolation in the differential diagnosis, but should be interpreted in the proper context, taking into consideration the patient's clinical history, the morphological appearance of the tumor, and judicious use of immunohistochemical markers. As another clue its nonspecificity CD10 immunoreactivity has also been noted in uterine leiomyosarcomas [7, 16].

CD10 expression of UES of the ovary is not well characterized. The previously published data are mainly available on uterine high-grade ESS. In such tumors, McCluggage and Sumathi [9] observed positive staining in four of six cases in a usually focal pattern. In their study on Müllerian system-derived neoplastic mesenchymal cells Mikami and Hata [17] noted moderate staining intensity in the single case of uterine high-grade endometrial sarcoma.

There are several aspects that need to be considered in the differential diagnosis of the presented case. UES of the ovary should be diagnosed only after excluding an undifferentiated carcinoma, malignant mixed Müllerian tumor or carcinosarcoma, respectively, and high-grade myogenic sarcoma. Therefore, extensive sampling to exclude skeletal or smooth muscle differentiation or even small foci of carcinoma is mandatory [7]. Recently, Soslow and Ali noted that the immunophenotype of most Müllerian adenosarcomas resembled that of endometrial stromal tumors (positive for estrogen and progesterone receptors, WT1, and CD10, with variable expression of smooth muscle markers, androgen receptor and cytokeratin); sarcomatous overgrowth was related to loss of expression of CD10 as well as estrogen and progesterone receptors [18]. Since there was no evidence for an expression of myogenic markers (desmin) and cytokeratin (AE1/AE3) by immunohistochemistry, and there was no condensation of tumor cells around endometriotic glands, we did not consider the presented case as a Müllerian adenosarcoma with stromal overgrowth. The lack of any epithelial differentiation as well as any AE1/AE3 cytokeratin immunoreactive cells excluded the diagnosis of carcinosarcoma.

Kurihara and Oda recommended a new terminology and classification of non-low-grade endometrial sarcomas [19]. They divided these sarcomas morphologically into undifferentiated endometrial sarcomas with nuclear uniformity (UES-U) and undifferentiated endometrial sarcomas with nuclear pleomorphism (UES-P). They reported on that UES-U share some molecular genetic and immunohistochemical characteristics with low-grade ESS, but that UES-P considerably differs from low-grade ESS. Morphology as well as low and focal estrogen and progesterone receptor immunoreactivity assign our case as UES-P. However, transition to areas with rather monotonous cells as noted in this case may indicate a link between UES-P and UES-U by a possible dedifferentiation of the latter component (Figures 1 and 2). 

Since this case of ovarian UES infiltrated the intestines, the possibility of a gastrointestinal stromaltumor (GIST) must be considered. Indeed, a recent study by Irving and Lerwill reported on gastrointestinal stromal tumors metastatic to the ovary [20]. These authors considered ESS in their differential considerations, too. Since most of the tumors in that study were misdiagnosed initially, the authors emphasized the importance of the distinction of ESS and GIST due to significant therapeutic and prognostic implications. In accordance with their observations, the case at hand had a negative immunophenotype for c-kit (CD117), which is considered a marker for GIST.

ESS metastatic from the uterus must be excluded before giving a diagnosis of primary ovarian ESS or UES, respectively [4]. The patient presented in this paper had hysterectomy seven years ago. Review of the corresponding slides did not show any evidence of a uterine stromal tumor.

In conclusion, CD10 immunoreactivity must be interpreted with caution since CD10 is frequently expressed in different gynecopathological as well as other lesions and, thus, nonspecific. Sarcomatous overgrowth of Müllerian adenosarcoma and high-grade leiomyosarcoma is important entities entering the differential diagnosis. Morphological features like association with ovarian endometriosis in this case, extensive sampling and appropriate immunohistochemistry including markers for cytokeratins and myogenic differentiation are mandatory to arrive at the correct diagnosis. Based on the recent literature and the findings in this case, CD10 immunoexpression is of no diagnostic value and not indicative as evidence for endometrioid stromal differentiation. UES should be considered as a high-grade sarcoma with no specific differentiation [7].
